# Infectious complications after transrectal MRI-targeted and systematic prostate biopsy

**DOI:** 10.1007/s00345-022-04104-1

**Published:** 2022-08-05

**Authors:** Inari Kalalahti, Kaisa Huotari, Andrew. M. Erickson, Anssi Petas, Hanna Vasarainen, Antti Rannikko

**Affiliations:** 1 Department of Urology, University of Helsinki and Helsinki University Hospital, Helsinki, Finland; 2 Research Program in Systems Oncology, Faculty of Medicine, University of Helsinki, Helsinki, Finland; 3 Department of Infectious Diseases, Helsinki University Hospital, University of Helsinki, Helsinki, Finland; 4 Nuffield Department of Surgical Sciences, University of Oxford, Oxford, UK; 5 Helsinki University Hospital, Hyvinkää Hospital, Sairaalakatu 1, 05850 Hyvinkää, Finland

**Keywords:** Infection, Complication, Targeted, Transrectal, Perineal, Prostate biopsy

## Abstract

**Purpose:**

To compare infectious complications after transrectal systematic prostate biopsy (SB) and magnetic resonance imaging (MRI)-targeted biopsy (TB) in a large retrospective cohort to assess whether one technique is superior to the other regarding infectious complications.

**Methods:**

A total of 4497 patients underwent 5288 biopsies, 2875 (54%) SB and 2413 (46%) MRI-TB only. On average, 12 SB cores and 3.7 MRI-TB cores were taken per biopsy session during the study period. Infection-related complications within 30 days were compared. The primary endpoint was a positive urine culture. Secondary endpoints were positive blood cultures, urine tests with elevated leukocytes ≥ 100 E6/L and elevated C-reactive protein (CRP) ≥ 100 mg/L. Chi-square test was used to compare the cohorts.

**Results:**

Positive urine cultures were found in 77 (2.7%) after SB and in 42 (1.7%) after MRI-TB (*p* = 0.022). In total, 46 (0.9%) blood culture positive infections were found, 23 (0.9%) occurred after SB and 23 (1.0%) after MRI-TB, (*p* = 0.848). Urine tests with elevated leukocytes ≥ 100 E6/L were found in 111 (3.9%) after SB and in 61 (2.5%) after MRI-TB (*p* = 0.006). Elevated CRP ≥ 100 mg/L was found in 122 (4.2%) after SB and in 72 (3.0%) after MRI-TB (*p* = 0.015). Blood cultures were drawn more often after SB than after MRI-TB, but the difference was not statistically significant. However, urine cultures and CRP were taken more often after SB than MRI-TB.

**Conclusion:**

Blood culture positive infections were equally rare after SB and MRI-TB. However, all other infectious complications were more common after SB than MRI-TB.

## Introduction

Prostate biopsy (PB) is a key step in the diagnosis and follow-up of prostate cancer (PC) and is the only way to obtain histopathological diagnosis in the absence of operative procedures such as radical prostatectomy or transurethral resection of the prostate. PB is also performed repeatedly during active surveillance. Although PB is generally considered a safe procedure, it can be associated with complications. A recent systematic review found bleeding to be the most common complication [1]. Infections after PB vary from mild lower urinary tract infections to febrile bacteremia and urosepsis. Infection rates after transrectal biopsy range from 0.1 to 7.0% and sepsis rates range from 0.3 to 3% [2, 3]. Hospitalization rates due to infections after PB are rising globally [4, 5]. Transperineal biopsy has been shown to significantly reduce infections compared to transrectal biopsy [6], but because of the need for anesthesia transrectal biopsy is still used in many clinics. Antibiotic prophylaxis is recommended as standard of care for all patients but antibiotic overuse has led to increasing antibiotic resistance [7], and as a consequence the most common microbe to cause infection after PB is fluoroquinolone-resistant Escherichia coli [8, 9].

Magnetic resonance imaging (MRI) and MRI-targeted biopsy (MRI-TB) improves the detection of PC compared to SB [10, 11]. Although smaller studies have reported on infections complications after MRI-TB, to our knowledge no studies have done so in a larger setting. In this study, we analyzed infectious complications after transrectal SB and MRI-TB occurring within 30 days of the procedure. In this setting, we aimed to establish if one technique is superior to the other regarding infections.

## Patients and methods

All patients who had undergone PB at Helsinki University Hospital between January 1, 2015 and September 5, 2019 were retrospectively entered into the study. Infection-related laboratory data within 30 days of the biopsy were obtained from the hospital laboratory database and compared between SB and MRI-TB. The obtained test results were: positive urine cultures, positive blood cultures, urine tests with elevated leukocytes ≥ 100 E6/L and elevated C-reactive protein (CRP) ≥ 100 mg/L. We also compared the frequency of tests taken as this reflects the suspicion of infection: blood cultures drawn (even if negative), urine cultures taken (even if negative) and CRP tests taken (even if not elevated).

The final dataset included 4497 patients and 5288 biopsy procedures. Out of these, 2875 (54%) were SB and 2413 (46%) were MRI-TB only (i.e., no concomitant SB taken). All biopsies were transrectal biopsies. In MRI-TB, no concomitant SB is routinely taken at our center. SB patients always received 12 cores, while MRI-TB patients received 3.7 cores on average. Most MRI-TB patients had only one suspicious lesion on MRI: 83% had one lesion, 15% had two lesions, and 2% had three lesions. MRI-TB was performed using a software-guided system (UroNav; Philips Healthcare, Best, The Netherlands). The medical charts of all patients with blood culture positive infections were reviewed to verify the source of infection. Three cases were identified as not being related to biopsy (a diverticulitis, an erysipelas and one infection related to catheterization) and were excluded from the analyses.

Antibiotic prophylaxis at our institution at the time of the study was ciprofloxacin 750 mg p.o. 1 h before biopsy. If the patient has traveled abroad within three months, fosfomycin trometamol 3 g p.o. was used instead of ciprofloxacin.

Chi-square test was used to compare the cohorts. Data were analyzed using R Statistical Software (version 4.0.5, Foundation for Statistical Computing, Vienna, Austria). The cutoff level for statistical significance was set at *p* < 0.05 for all tests.

## Results

**Fig. 1:**
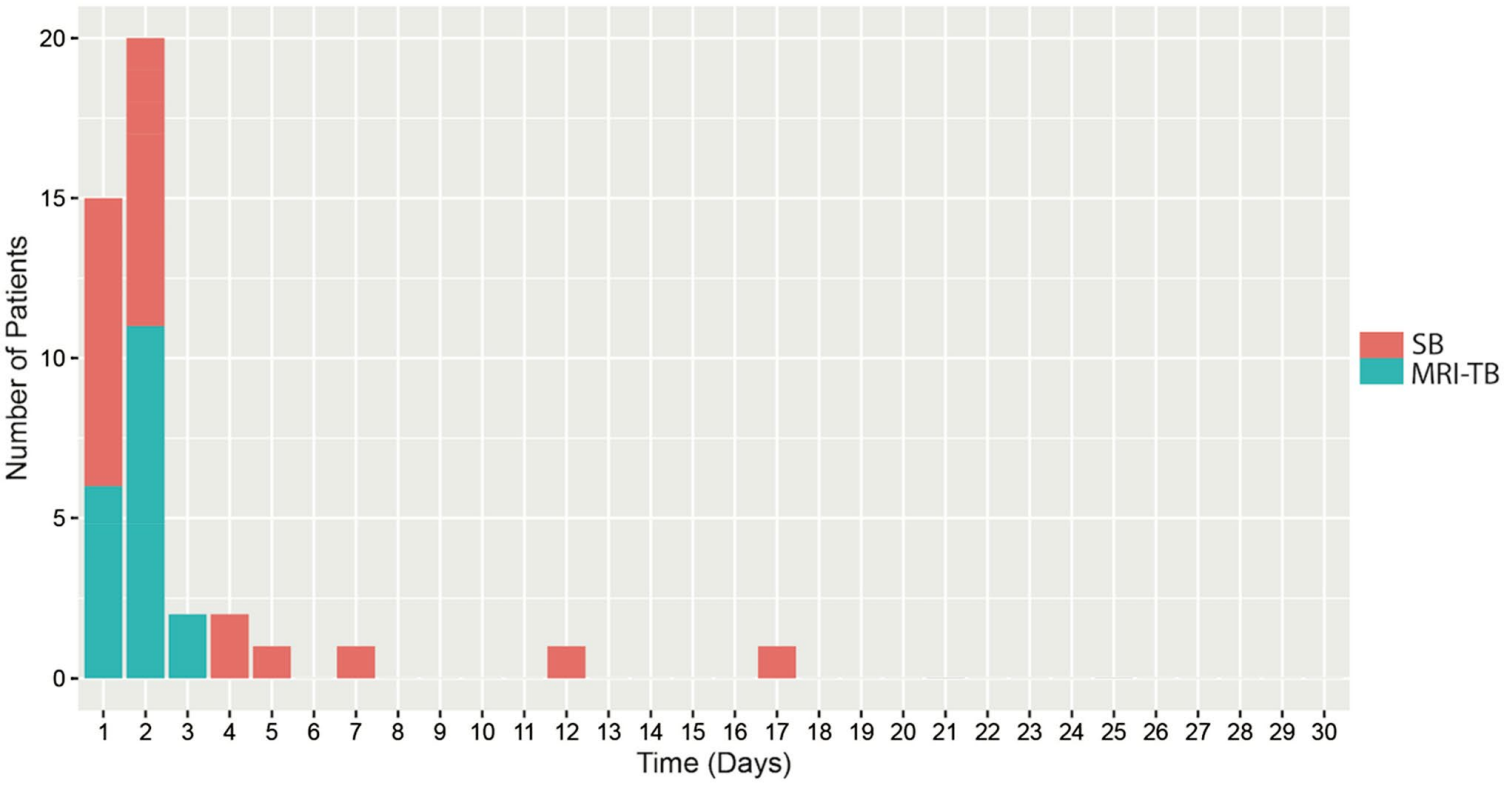
Blood culture positive infections after biopsy in the cohorts

**Fig. 2:**
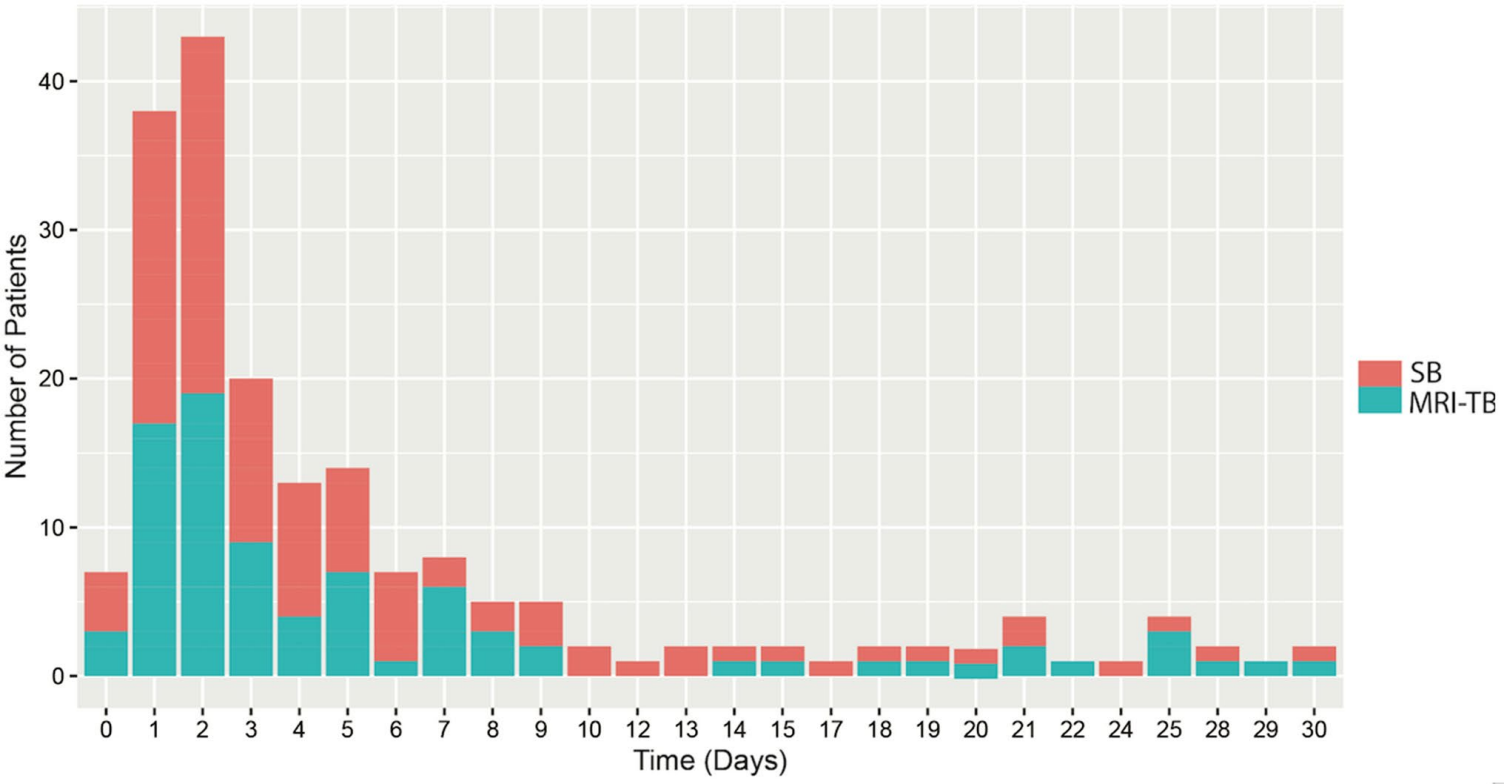
Blood cultures drawn after prostate biopsy in the cohorts

Blood culture positive infections were rare in our data. In the entire cohort, 46 (0.9%) blood culture positive infections were identified. Out of these, 23 (0.9%) occurred after SB and 23 (1.0%) after MRI-TB (p = 0.848). Most infections occurred or were suspected within the first few days after biopsy (Figs. 1 and 2).

For all of the other endpoints, there was a statistically significant difference in favor of MRI-TB (Table 1). Positive urine cultures were found in 77 (2.7%) after SB and in 42 (1.7%) after MRI-TB (*p* = 0.022). Urine tests with elevated leukocytes ≥ 100 E6/L were found in 111 (3.9%) after SB and in 61 (2.5%) after TB (*p* = 0.006). CRP ≥ 100 mg/L was found in 122 (4.2%) after SB and in 72 (3.0%) after TB (*p* = 0.015).

**Table 1:** Infectious complications in the cohorts

	Systematic biopsy	MRI-targeted biopsy	*p* value
Blood culture positive infection, *N* (%)	23 (0.9)	23 (1.0)	0.848
Positive urine culture, *N* (%)	77 (3)	42 (2)	0.022
Urine test with elevated leukocytes ≥ 100 E6/L, *N* (%)	111 (4)	61 (3)	0.006
CRP ≥ 100 mg/L, *N* (%)	122 (4)	72 (3)	0.015
Blood cultures drawn, *N* (%)	106 (4)	85 (4)	0.750
Urine cultures taken, *N* (%)	328 (11)	206 (9)	< 0.001
CRP taken, *N* (%)	403 (14)	246 (10)	< 0.001

*CRP*  C-reactive protein, *MRI*  magnetic resonance imaging

For the tests taken, CRP and urine cultures were taken significantly more often after SB than MRI-TB. Blood cultures were drawn more often after SB than after MRI-TB, but the difference was not statistically significant (Table 1).

## Discussion

Signs and symptoms of infectious complications were more common after SB than MRI-TB. We did not find a statistically significant difference between the cohorts in blood culture positive septic infections, the most feared form of infectious complication. This was expected and reflects the low number of events, i.e., lack of statistical power for this. As all other infectious complications, surrogates, were more common after SB, it suggests that a difference in bacteremic infections may exist as well. In order for us to have detected a difference of 0.8% vs 1.2% with the power of 0.80, we would have needed almost 8000 patients per group. Interestingly, a randomized trial was recently launched in the USA to compare transrectal biopsies to transperineal biopsies for a “change in infection adverse events” with a planned recruitment of 1302 participants [12].

In transrectal biopsy, the needle punctures the bowel wall and may spread bacteria on its route to the prostate. In MRI-TB, 3 to 5 cores are usually taken per lesion. Since most patients only have one lesion, the number of cores needed is much lower in MRI-TB than in SB where 12 cores are taken per patient. In our study, typical signs of urinary tract infections, positive urinary cultures, elevated CRP and leucocytes in urine were all more common after SB than MRI-TB. The CRP test is not specific to urogenital infections and it is possible that some observed CRP elevations were due to other infections and may not be biopsy-related. However, the timeline of the CRP elevation coincided with the biopsy. If a patient is experiencing symptoms of urinary tract infections after biopsy urine cultures and CRP tests are common investigations. Both urine cultures and CRP tests were taken more often after SB. This supports the assumption that infectious complications are more common after SB.

The risk of bleeding and pain after PB has been associated to an increasing number of biopsy cores [13, 14]. A similar relationship has not clearly been seen for infections. A recent systematic review did not find an association between the number of biopsy cores and infectious complications [15]. However, a multicenter RCT comparing different biopsy techniques found that an increase in the cores taken led to an increase in infectious complications, although the difference was not statistically significant [16]. Our study demonstrates a relationship between the number of cores taken and increased infectious complications.

Several factors that may influence the risk of complications after PB have been studied. These include prior antibiotic treatment, foreign travel, comorbidities, the number of biopsy cores taken, number of injections for periprostatic nerve block, the type and size of biopsy needle and rectal enema before biopsy [15]. The risk of infection is lower in transperineal biopsy. In the transperineal approach, the biopsy needle avoids the rectal mucosa that is heavily contaminated by bacteria and cannot be fully disinfected. Because of this advantage, transperineal biopsy is now the recommended standard of care by the European Association of Urology guidelines [17]. The use of the transperineal technique is still limited because it typically requires anesthesia although transperineal biopsy in local anesthesia currently gaining more attention. In transperineal biopsy, the infection rate can be as low as 0.1% [6]. The sepsis rate in our study was 0.9%, but this could be lowered by additional methods such as povidone–iodine preparation, which was initiated at our center after the study period.

A limitation of this study is the retrospective design. This limitation is partially balanced by our large dataset. As blood culture positive infections occur at a very low rate, a large dataset is essential in studying them. Despite our large dataset, our study was underpowered for post-biopsy septic infections that in our center occur at a relatively low rate.

Based on our results, we advocate a biopsy strategy where less cores are taken and to omit SB when MRI-TB is applicable. Our data suggest a correlation between the number of biopsy cores and increased likelihood of infection. Transperineal is also a sensible method to further reduce prostate biopsy-related infections.
